# Gender specific aspects of digital screening for atrial fibrillation: insights from the randomized eBRAVE-AF trial

**DOI:** 10.1093/ehjdh/ztaf071

**Published:** 2025-06-19

**Authors:** Luisa Freyer, Peter Spielbichler, Lukas von Stülpnagel, Konstantinos Mourouzis, Lukas Tenbrink, Laura Elisa Villegas Sierra, Maria F Vogl, Lauren E Sams, Annika Schneidewind, Mathias Klemm, Steffen Massberg, Axel Bauer, Konstantinos D Rizas

**Affiliations:** Medizinische Klinik und Poliklinik I, University Hospital Munich, Ludwig-Maximilians University, Munich Ziemssenstrasse 5, Munich 80336, Germany; German Center for Cardiovascular Research (DZHK), partner Site: Munich Heart Alliance, Munich, Germany; Medizinische Klinik und Poliklinik I, University Hospital Munich, Ludwig-Maximilians University, Munich Ziemssenstrasse 5, Munich 80336, Germany; Medizinische Klinik und Poliklinik I, University Hospital Munich, Ludwig-Maximilians University, Munich Ziemssenstrasse 5, Munich 80336, Germany; Medizinische Klinik und Poliklinik I, University Hospital Munich, Ludwig-Maximilians University, Munich Ziemssenstrasse 5, Munich 80336, Germany; Medizinische Klinik und Poliklinik I, University Hospital Munich, Ludwig-Maximilians University, Munich Ziemssenstrasse 5, Munich 80336, Germany; Medizinische Klinik und Poliklinik I, University Hospital Munich, Ludwig-Maximilians University, Munich Ziemssenstrasse 5, Munich 80336, Germany; Medizinische Klinik und Poliklinik I, University Hospital Munich, Ludwig-Maximilians University, Munich Ziemssenstrasse 5, Munich 80336, Germany; Medizinische Klinik und Poliklinik I, University Hospital Munich, Ludwig-Maximilians University, Munich Ziemssenstrasse 5, Munich 80336, Germany; German Center for Cardiovascular Research (DZHK), partner Site: Munich Heart Alliance, Munich, Germany; Medizinische Klinik und Poliklinik I, University Hospital Munich, Ludwig-Maximilians University, Munich Ziemssenstrasse 5, Munich 80336, Germany; Medizinische Klinik und Poliklinik I, University Hospital Munich, Ludwig-Maximilians University, Munich Ziemssenstrasse 5, Munich 80336, Germany; German Center for Cardiovascular Research (DZHK), partner Site: Munich Heart Alliance, Munich, Germany; Medizinische Klinik und Poliklinik I, University Hospital Munich, Ludwig-Maximilians University, Munich Ziemssenstrasse 5, Munich 80336, Germany; German Center for Cardiovascular Research (DZHK), partner Site: Munich Heart Alliance, Munich, Germany; University Hospital for Internal Medicine III, Medical University of Innsbruck, Innsbruck, Austria; Medizinische Klinik und Poliklinik I, University Hospital Munich, Ludwig-Maximilians University, Munich Ziemssenstrasse 5, Munich 80336, Germany; German Center for Cardiovascular Research (DZHK), partner Site: Munich Heart Alliance, Munich, Germany

**Keywords:** eBRAVE-AF, Atrial fibrillation, Oral anticoagulation, Digital screening, Photoplethysmography, Sex-differences

## Abstract

**Aims:**

Smartphone-based digital screening was shown to increase the detection rate of atrial fibrillation (AF) requiring oral anticoagulation (OAC) compared with usual care. In this pre-specified subgroup analysis of the eBRAVE-AF trial, we explored sex-specific differences in digital AF-screening.

**Methods and results:**

In eBRAVE-AF (NCT04250220), participating policyholders of a German health insurance company were randomly assigned to a 6-month digital or conventional AF-screening strategy. For digital screening, participants used smartphone-based photoplethysmography (PPG) to detect pulse wave irregularities, which were confirmed using 14-day external ECG-recorders. The primary endpoint was newly diagnosed AF treated with OAC. After 6 months, participants were assigned to a second, cross-over study-phase. The efficacy of AF-screening in women and men was assessed by Cox-regression analysis. 5551 (31% females; 55% ≥ 65 years) of 67 488 invited policyholders free of AF participated in the study and were randomly assigned to digital screening (*n* = 2860) or usual care (*n* = 2691). Participation rate was significantly higher among men than women (8.7% vs. 7.3%; *P* < 0.001). Male sex was a significant predictor for reaching the primary endpoint (HR 1.74; 95% CI: 1.08–2.82, *P* = 0.023), which was pronounced in patients undergoing digital screening (HR 2.48; 95% CI: 1.52–4.05, *P* < 0.001). Digital screening did not significantly increase the detection rate of AF requiring OAC in women (HR 1.83; 95% CI: 0.74–4.54; *P* = 0.193; *P*-interaction = 0.563).

**Conclusion:**

Men showed higher willingness to participate in this digital study and digital AF-screening was effective for them. While digital screening increased the detection rate of AF with OAC in women, the effect was not statistically significant, likely due to limited power.

## Graphical Abstract

**Graphical Abstract:**
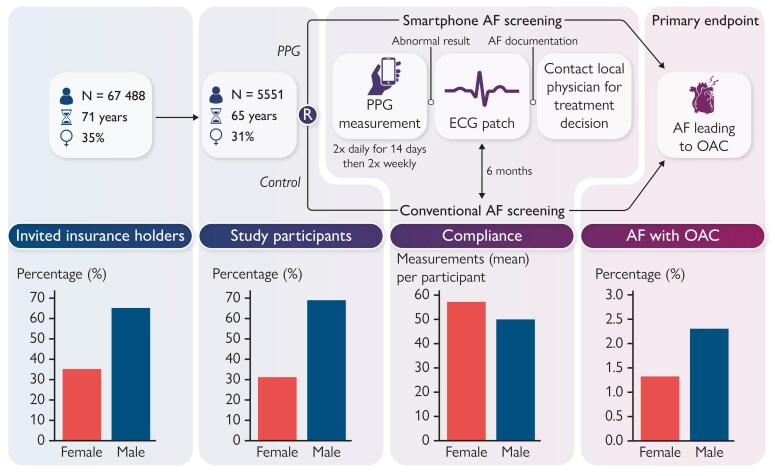

## Introduction

Atrial fibrillation (AF) is the most common sustained arrhythmia and continues to make a progressive and substantial impact on public health. Currently, AF affects ∼60 million people worldwide.^1^ AF is associated with significant sex differences in prevalence, clinical manifestation, risk factors, comorbidities, treatment and outcomes.^2–4^ Sex and age are the most powerful predictors of AF. After adjusting for age, the incidence of AF in men is 1.5 to 2 times higher than women.^5^ While AF is more prevalent in men, women with AF are usually more symptomatic and have higher risk for developing complications, including stroke, heart failure^5^ and death.^6^

For timely detection of AF, current guidelines recommend opportunistic screening by pulse taking or ECG-guided monitoring in both men and women aged ≥ 65 years.^6^ Besides opportunistic AF screening, consumer-initiated AF screening using smart devices, has become increasingly prevalent. Smart devices with optical sensors, such as smartphones and smartwatches, can detect irregularities in the pulse wave sequence, which established them as valuable screening tools in the broad population. Three large-scale observational studies,^7–9^ demonstrated the feasability of digital AF screening, using a ‘direct-to-consumer’ approach, by including owners of wrist-worn devices. The eHealth-based bavarian alternative detection of Atrial Fibrillation trial (eBRAVE-AF) was the first siteless randomized trial to assess the efficacy of a smart-device based screening strategy to detect treatment-relevant AF in direct comparison with conventional symptom-based screening. Digital screening more than doubled the detection rate of treatment-relevant AF compared with usual care.^10^

In this pre-specified subgroup analysis of eBRAVE-AF, we aimed to investigate the effect of sex on participation rate, compliance and efficacy of digital screening.

## Methods

The present study was a pre-specified substudy of the eBRAVE-AF trial. The design and main results of eBRAVE-AF have been previously published.^11^ eBRAVE-AF (NCT04250220) was an academic, prospective, randomized, siteless, open-label digital trial with cross-over design, sponsored by the Ludwig-Maximilians University hospital Munich, Germany. It was powered to assess the efficacy of digital, smart-device-based AF screening compared with usual care for detection of treatment-relevant AF in an elderly at-risk population. The eBRAVE-AF study protocol was approved by the medical ethics committee of the Ludwig-Maximilians University, Munich, Germany (#18-779). All participants were provided informed consent. The study protocol and statistical analysis plan of the trial are included in the Supplementary material online of the main results publication.

### Trial design and study participants

Study participants were recruited from the pool of policyholders of ‘Versicherungskammer Bayern’, a German private health insurance company. Individuals aged 50–90 years with a CHA_2_DS_2_-VASc score of ≥1 in men or ≥2 in women with no prior history of AF, or treatment with oral anticoagulation (OAC), were eligible. 67 488 policyholders met the inclusion criteria and were invited to participate by regular mail. The enrolment process was conducted purely digital without in-person patient contact. No reminder was sent to policyholders not responding to the initial invitation. The trial enrolled a total of 5551 participants from February 2020 until July 2020, who downloaded a specifically designed study app (eBRAVE-AF app, design-IT GmbH) on their smartphone and provided electronic informed consent. The eBRAVE-AF app handled the communication with the participants.

### Randomisation and cross-over

**Figure 1:**
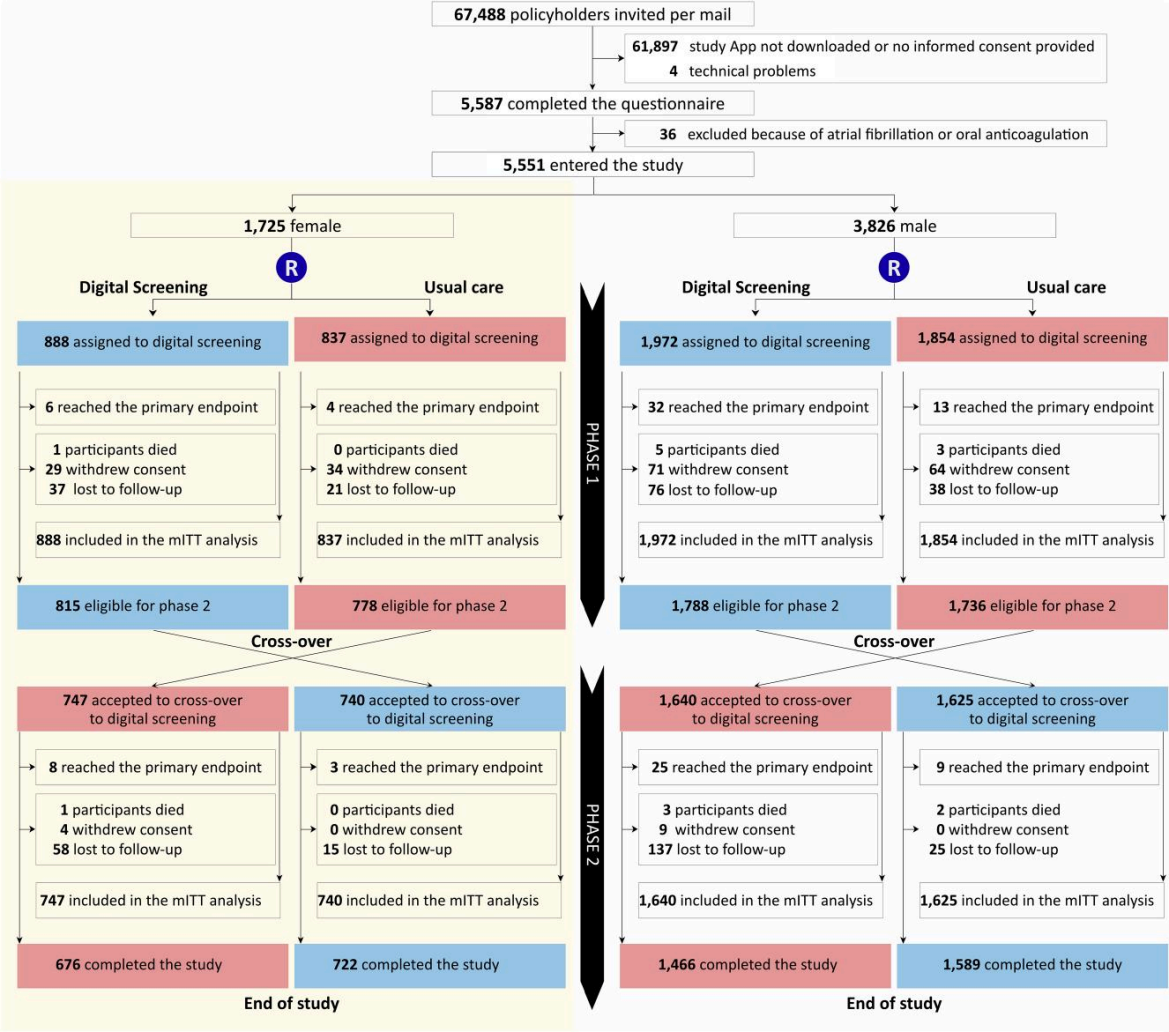
Consort diagram.

The study was designed as a parallel-group randomized trial with a subsequent cross-over for secondary analyses. After providing informed consent, participants triggered a simple randomisation process in the eBRAVE-AF app, by which they were assigned to either an e-health based strategy (Group 1) or usual care (Group 2). After 6 months, participants who did not reach the primary endpoint in the first study phase, were still alive, and did not withdraw informed consent, were invited to participate in a second 6-month study phase with cross-over assignment of Group 1 to usual care and Group 2 to digital screening (*Figure 1*).

### Digital AF screening and usual care

Digital AF screening consisted of repetitive 1-min photoplethysmography (PPG) pulse wave self-measurements using a smartphone app (Preventicus Heartbeats, Preventicus) on their own smartphones (Android and iOS), which was, in case of an abnormal PPG result, followed by a confirmatory ECG recording by means of a 14-day external ECG loop recorder (CardioMem CM 100XT, GETEMED). The Preventicus Heartbeats app is a CE-certified Class 2a medical device for the detection of AF. Participants were instructed to perform PPG measurements twice daily for the first 14 days and twice weekly thereafter (weeks 3–26). In case of a confirmed abnormal PPG finding, participants were advised to consult their local treating physicians, not being involved in the study, who made the final decision regarding treatment, such as initiation of OAC.

Usual care aimed to map the natural history of AF detection and therefore, no study-related diagnostic procedures were performed.

### Data acquisition

The proportion of females among all policyholders was obtained from the health insurance company and refers to year 2025. Anonymized baseline data of invited insurance holders, which for regulatory reasons were only available for 51.775 of 67.488 invited individuals (77%), were obtained from claims data. For all 5551 study participants baseline characteristics were derived from claims data and personal questionnaires. Clinical and follow-up information of study participants were collected via in-app questionnaires or by phone and there was no in-person contact with the study participants throughout the study. Source documents were requested for all suspected endpoints.

### Study endpoints

The primary efficacy endpoint was newly diagnosed AF that led to new prescription of OAC by an independent physician. For the pooled-analysis that was used in this substudy the endpoint was evaluated 12 months after randomisation. Secondary endpoints were newly diagnosed AF, newly prescribed OAC, stroke, thromboembolic events and major bleedings Bleeding Academic Research Consortium (BARC ≥ 2). Major adverse cardiac or cerebrovascular events (MACCE) were defined as the composite of stroke, pulmonary artery embolism, deep vein thrombosis, hospitalisation for acute decompensated heart failure and myocardial infarction. All endpoints were assessed by app-based questionnaires and telephone calls as well as by insurance claims data (ICD) from all participants at the end of the study. For all suspected primary endpoints, source documents including medical reports and hospital records, were requested. If source data were not available, the endpoint documented by ICD (ICD-10-CM and ATC codes) was considered to be correct. The primary endpoint was adjudicated by an independent endpoint committee blinded to the study group allocation.

### Statistical analysis

Continuous data are presented as medians with lower and upper quartiles and were compared using Wilcoxon test. Categorical data are summarized with the use of frequencies and proportions and were compared using the chi-square test. Differences in the proportion of women between insured and invited policyholders, as well participants were examined using chi-square test. Outcomes were analyzed using time-to-event methods. Event rates and cumulative proportions were estimated using the Kaplan-Meier method with 95% CI calculated based on Greenwood’s method and were compared using log-rank statistics. Cox proportional hazards regression was utilized as a time-to-event analysis. Male sex, diabetes, hypertension, stroke, vascular disease and heart failure were reported as categorical variables, age was reported as a continuous variable for the purpose of uni- and multivariate analysis. The effect of the mode of screening on the primary endpoint was primarily tested using Cox regression analysis. The two phases of the study were combined using group-assignment as time-dependent co-variate. The window-phase between the ending of the first phase of the study and the cross-over assignment to the reverse-group, which was excluded from the analysis in the original publication, was considered as prolongation of the first phase of the study. The effect of digital screening on outcome for men and women was assessed using Cox-regression models and inclusion of the product of digital screening grouping-variable with sex as co-variate in the statistical model. All endpoints were censored 12 months after signing the electronic informed consent. Analyses were done on a modified intention-to-treat basis according to the protocol, in which patients who had known AF or an ongoing treatment with OAC were excluded. For all analyses, differences were considered statistically significant when the two-sided *P* value was < 0.05. All statistical analyses were performed using CRAN R version 4.3.1.

## Results

### Patient characteristics

The pool of policyholders of our collaborating health care insurance company ‘Versicherungskammer Bayern’ consists of 57.9% men and 42.1% women. Based on the inclusion criteria of the trial 65% of the invited policyholders were males and 35% females (*P* < 0.001 for the ratio of invited vs. non-invited female policyholders). Out of the invited policyholders (median age 71, IQR 64–78 years), 5551 individuals agreed to participate and were included in the trial (31% females, median age 65, IQR 60–71 years). The participation rate of invited policyholders was 7.3% in women and 8.7% in men (*P* < 0.001).

Out of the 5551 participants, 2860 (888 female and 1972 male participants) were assigned to the digital screening and 2691 (837 female and 1854 male participants) to the usual care group (*Figure 1*). After 6 months follow-up 5117 participants (1593 female and 3524 male participants) were invited to enter the second cross-over phase of the study. A total of 4752 participants (1487 female and 3265 male participants) accepted the invitation and were assigned to the reverse study groups. After combining the two phases of the study 5247 (95%) participants (1635 female and 3612 male participants) were assigned to digital monitoring during the entire study and 5056 (91%) participants (1577 female and 3579 male participants) were assigned to usual care.

*Table 1* depicts patients’ characteristics stratified by sex. Male participants were older, had a higher BMI and suffered more frequently from cardio- and cerebrovascular comborbidities including coronary artery disease (18.2% vs. 7.0%, *P* < 0.001), peripheral artery diseases (1.9% vs. 0.9%, *P* = 0.008) and strokes (6.7% vs. 3.8%, *P* < 0.001). Accordingly, male participants were characterized by a higher cardiovascular risk profile including higher prevalence of diabetes mellitus (14.8% vs. 8.8%, *P* < 0.001) and arterial hypertension (67.1% vs. 61.5%, *P* < 0.001). In contrast, female participants reported more symptoms such as dyspnoe and angina (28.3% vs. 16.8% *P* < 0.001) or palpitations (29.2% vs. 16.5%, *P* < 0.001) and were more frequently treated with beta-blockers (6.1% vs. 1.5%, *P* < 0.001).

**Table 1:** Characteristics and medical treatments of the study participants stratified sex

	Females (*N* = 1725)	Males (*N* = 3826)	*P*-value
**Characteristics**			
Age (years)	65 (59–70)	66 (60–71)	0.001
BMI (kg/m²)	25 (23–39)	27 (25–30)	<0.001
CHA_2_DS_2_-VASc score	3 (3–4)	3 (2–3)	<0.001
Coronary heart disease	120 (7.0)	698 (18.2)	<0.001
Previous MI	34 (2.0)	328 (8.6)	<0.001
Heart failure	80 (4.6)	146 (3.8)	0.174
Valvular heart disease	129 (7.5)	217 (5.7)	0.012
Peripheral artery disease	15 (0.9)	71 (1.9)	0.008
History of stroke	65 (3.8)	255 (6.7)	<0.001
COPD	5 (0.3)	9 (0.2)	0.930
Chronic kidney disease	6 (0.3)	10 (0.3)	0.774
Diabetes	151 (8.8)	566 (14.8)	<0.001
Hypertension	1061 (61.5)	2558 (67.1)	<0.001
History of loss of consciousness	102 (5.9)	177 (4.6)	0.050
History of bleeding	56 (3.2)	161 (4.2)	0.101
History of thromboembolism	92 (5.3)	342 (8.9)	<0.001
**Symptoms**			
Dyspnea or angina	489 (28.3)	643 (16.8)	<0.001
Palpitations	504 (29.2)	630 (16.5)	<0.001
**Medication**			
ACE inhibitor or ARB	228 (13.2)	502 (13.1)	0.949
Beta-blocker	105 (6.1)	59 (1.5)	<0.001
Aspirin	12 (0.7)	25 (0.7)	0.998
Other antiplatelet agents	4 (0.2)	3 (0.1)	0.279

ACE, angiotensin converting enzyme; ARB, angiotensin receptor blocker; BMI, body mass index; COPD, Chronic obstructive pulmonary disease; ICD, implantable cardioverter-defibrillator; MI, myocardial infarction.

### Follow-up

Within 12 months, 67 female participants (3.9%) and 144 male participants (3.8%) withdrew consent (*P* = 0.888). Moreover, 131 female participants (7.6%) and 276 male participants (7.2%) were lost to follow-up (*P* = 0.655).

### Detection of treatment-relevant AF

**Figure 2:**
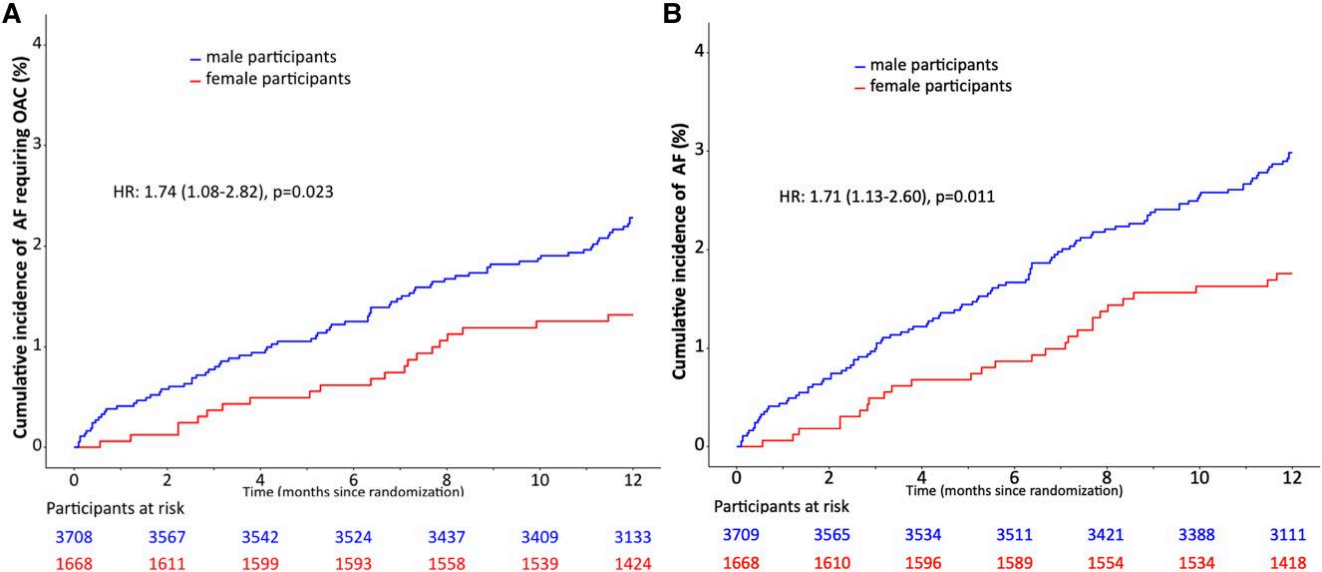
Cumulative detection rates of atrial fibrillation requiring oral anticoagulation (*A*) and atrial fibrillation (*B*) stratified by sex (HR: hazard ratio).

**Figure 3:**
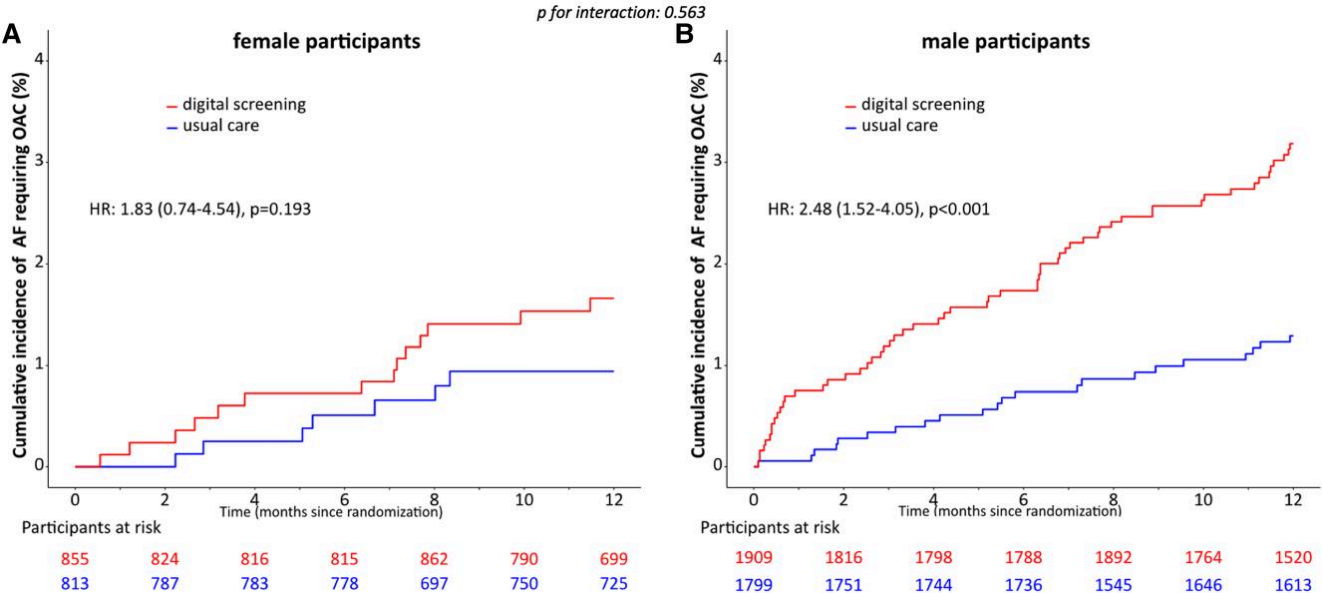
Cumulative detection rates of atrial fibrillation requiring oral anticoagulation in female participants (*A*) and male participants (*B*) stratified by screening method (HR: hazard ratio).

Within a follow-up period of 12 months including both phases of the study and both arms (digital screening and usual care), AF was newly diagnosed in 106 of the 3826 male participants (cumulative event rate 3.0%; 95% CI: 2.4–3.5%) with prescription of OAC in 81 participants (2.3%; 95% CI: 1.8–2.8%) and in 28 of 1725 female participants (cumulative event rate 1.8%; 95% CI: 1.1–2.4%) with initiation of OAC in 21 participants (1.3%; 95% CI: 0.8–1.9%). In the total population, male sex was a significant predictor for reaching the primary endpoint (HR 1.74; 95% CI: 1.08–2.82; *P* = 0.023; *Figure 2A*), as well as the secondary endpoint of newly diagnosed AF (HR 1.71; 95% CI: 1.13–2.60; *P* = 0.011; *Figure 2B*). In multivariable analysis, male sex remained an independent predictor for newly diagnosed AF (1.61; 95% CI: 1.06–2.45; *P* = 0.025; see Supplementary material online, *Table 2*), but not for the primary endpoint (1.58; 95% CI: 0.97–2.56; *P* = 0.067; see Supplementary material online, *Table 1*). 76.4% of male participants and 75.0% of female participants were treated with OAC following AF-diagnosis (*P* = 1.000). Digital screening significantly increased the detection rate of AF requiring OAC in men (HR 2.48; 95% CI: 1.52–4.05, *P* < 0.001; *Figure 3A*). In women this effect did not reach the level of statistical significance (HR 1.83; 95% CI: 0.74–4.54, *P* = 0.193; *Figure 3B*). There was no significant interaction (*P* = 0.563) between sex and the efficacy of digital screening for detecting AF requiring OAC.

**Table 2:** Association of digital screening with the primary and secondary endpoints 1 year after randomisation stratified by sex

	Female participants				Male participants				Interaction
Endpoint	Events/Patients Digital screening	Events/Patients Usual care	Hazard ratio (95% CI)	*P*-value	Events/Patients Digital screening	Events/Patients Usual care	Hazard ratio (95% CI)	*P*-value	*P*-value
**Primary endpoint**	14/1602	7/1553	1.83 (0.74–4.53)	0.193	59/3550	22/3424	2.48 (1.52–4.05)	<0.001	0.562
**Secondary endpoints**									
Newly diagnosed AF	19/1601	9/1550	1.94 (0.88–4.28)	0.103	73/3546	32/3415	2.11 (1.39–3.20)	< 0.001	0.846
Newly diagnosed OAC	22/1600	11/1549	1.81 (0.88–3.73)	0.109	84/3544	42/3409	1.82 (1.26–2.65)	0.001	0.979
Ischaemic stroke	4/1604	4/1558	0.93 (0.23–3.72)	0.919	17/3553	19/3445	0.84 (0.44–1.61)	0.594	0.892
Thromboembolic events	4/1605	3/1558	1.19 (0.27–5.31)	0.821	21/3556	13/3443	1.45 (0.73–2.91)	0.288	0.810
Major bleeding	4/1603	3/1556	1.22 (0.27–5.48)	0.790	29/3550	19/3439	1.42 (0.80–2.54)	0.232	0.856
MACCE	11/1603	7/1555	1.44 (0.56–3.71)	0.454	56/3544	45/3425	1.15 (0.77–1.70)	0.494	0.667
Death	2/1606	1/1559	1.81 (0.16–20.0)	0.627	9/3561	6/3454	1.38 (0.49–3.88)	0.541	0.838

Number of patients includes the overall number of patients included in both phases of the trial. Patients reaching an endpoint during the first phase of the trial were not included in the second phase of the trial for this particular endpoint. Therefore, the number of patients at risk differs for the different endpoint.

AF, atrial fibrillation; MACCE, major adverse cardiac and cerebrovascular events; OAC, oral anticoagulation.

### Secondary endpoints

Throughout the study 41 women (2.6%; 95% CI: 1.8–3.3%) and 132 men (3.7%; 95% CI: 3.1–4.3%) recorded an abnormal PPG (HR 1.46; 95% CI: 1.03–2.07; *P* = 0.035). AF diagnosis after positive PPG was made in 9 female (0.6%; 95% CI: 0.2–0.9%) and 53 male participants (1.5%; 95% CI: 1.1–1.9%) cases, respectively (HR 2.67; 95% CI: 1.32–5.40; *P* = 0.006).

*Table 2* shows the association of sex with secondary endpoints. Digital screening significantly increased the detection rate of AF with and without initiation of OAC in men (HR 2.11; 95% CI: 1.39–3.20; *P* < 0.001). In women this effect did not reach the level of statistical significance (HR 1.94; 95% CI: 0.88–4.28; *P* = 0.103). The interaction between sex and the efficacy of digital screening for detecting AF with and without treatment with OAC was not statistically significant (*P* = 0.846). 15 male participants (0.4%) and 3 female participants (0.2%) died (HR 2.25; 95% CI: 0.65–7.76; *P* = 0.201 for the association between sex and death). The incidence of MACCE within 12 months was 2.8% (95% CI: 2.3–3.4%) in male participants and 1.1% (95% CI: 0.6–1.7%) in female participants (*P* < 0.001).

### Adherence to digital screening

**Figure 4:**
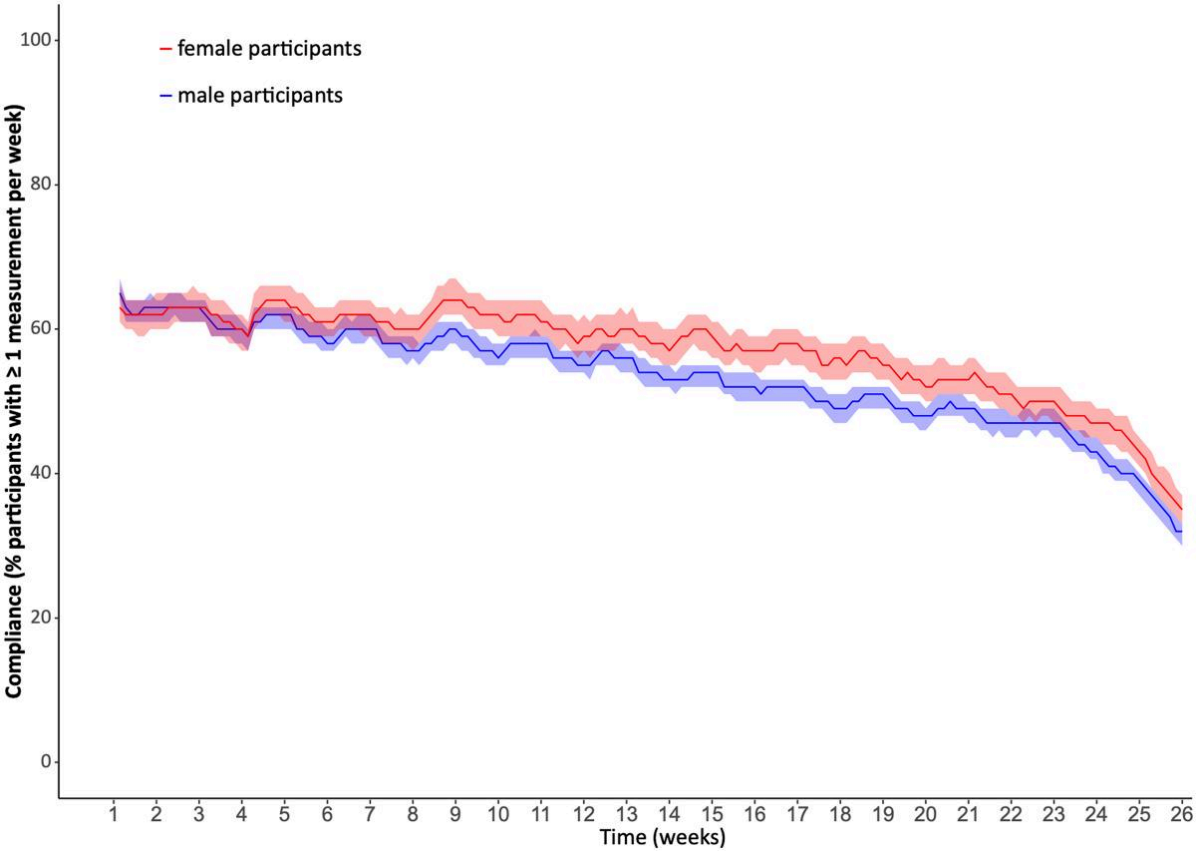
Adherence to study protocol as percentage of study participants with ≥ 1 photoplethysmography measurement per week.

During the study, 300 509 PPG-measurements were performed, corresponding to a median of 53 (IQR 14–76) per active participant. 1421 of 1635 female participants (86.9%) assigned to digital screening and 3172 of 3612 male participants (87.8%) assigned to digital screening performed at least one valid PPG measurement during the entire study (*P* = 0.357). Female sex was associated with a better compliance in terms of more frequent PPG-measurements (57; IQR 21–78), compared with male sex (50; IQR 13–75; *P* < 0.001). *Figure 4* depicts the percentage of female and male study participants who performed ≥1 PPG measurement per week during digital screening.

## Discussion

The usage of wearables and smartphones to monitor cardiac rhythm holds great potential for AF screening and early AF detection. The eBRAVE-AF study showed that digital, smartphone-based AF screening by intermittent PPG measurements approximately doubled the detection rate of treatment relevant AF, when compared with usual care.^10^ In this pre-specified substudy, we investigated differences between women and men in participation and effectiveness of digital screening for detecting AF requiring OAC. We found that men showed higher willingness for study participation and significantly benefited from digital screening with higher detection rates of AF requiring OAC. Females showed lower paticipation rates but high compliance to the study protocol. Digital screening did not significantly increase the detection rate of AF requiring OAC in female participants, most probably because of overall less primary endpoints among females.

Sex-specific subgroup analyses for effectiveness of digital AF screening are of great clinical importance because female patients show higher rates of AF-related complications such as myocardial infarction^5,12^ and death.^5,13^

Three previous consumer-oriented observational studies, the Apple heart, Fitbit heart and Huawei heart study, have evaluated the use of wearable devices for continuous AF screening in an impressive combined total of over 1 million participants, with female participation ranging between 13% and 70%.^7–9^ These studies targeted owners of brand-specific smart devices without prior risk-stratification, resulting in a relatively young median participant age of 35 to 47 years and consequently in a relatively low AF detection rate of 0.07%. Moreover, without a control group, the additional benefit of smart device based AF screening over usual care remained unclear. Since these trials used a direct-to-consumer enrolment approach, the variation of female participants is likely the result of sex differences in brand preference and device usage as well as sex-specific willingness to participate in digital AF screening studies. A recent trial on smart device ownership and digital health data sharing found 98% smartphone ownership with no sex-specific differences, but significantly higher wearable ownership among women compared with men (62% vs. 53%). Additionally, men demonstrated a greater willingness to share their digital health data for research purposes.^14^

In eBRAVE-AF, only 31% of study participants were females. The lower percentage of women can be attributed to several factors. First, there is previously documented underrepresentation of females in the German private health care insurance system.^15^ The pool of policyholders of our collaborating health care insurance company ‘Versicherungskammer Bayern’ consists of 57.9% men and 42.1% women. Second, in eBRAVE-AF there was a pre-selection for males based on the inclusion criteria of the trial. As the CHA_2_DS_2_-VASc score inclusion criterion was adapted for men and women (≥1 in men or ≥2 in women), the overall sex distribution was highly dependent on the sex-specific incidence of arterial hypertension, diabetes mellitus, stroke, vascular disease and congestive heart failure, which is more common in men than women.^16–20^ For this reason there was a statistically significant difference between female policyholders and policyholders who met our inclusion criteria and were ultimately invited to participate in our study. Finally, compared with males (8.7% participation rate), there was an overall significantly lower willingness of invited females to participate in eBRAVE-AF (7.3% participation rate). Consistent with our finding, a previous digital, siteless AF screening trial using a similar direct-to-participant recruitment process and a 14-day ECG patch for AF-screening reported lower participation among women.^21^ In contrast, another fully digital AF-screening trial with a more self-initiated enrolment process and two studies with traditional mail invitation followed by ECG screening at study centres reported higher participation among women.^22–25^

In our study, female study participants were younger and had fewer cardiovascular comorbidities, but presented more often with cardiorespiratory symptoms compared with men. Female participants also showed better compliance to the study protocol in terms of more frequent PPG measurements. Higher adherence to wearable use in female participants has also been reported in previous wearable trials.^26^ This finding is of great importance, as previous analyses showed a significant correlation between the frequency of PPG measurements and AF detection.^10^

Data on differences in AF detection rates between women and men are largely consistent across digital AF screening trials. In line with Apple heart study and Fitbit heart study, female participants in eBRAVE-AF showed lower rates of irregular heart rhythm reports and lower rate of digitally detected AF compared with male participants.^7,8^ These results seem plausible, as previous studies have shown a lower age-adjusted prevalence (7.4% in women, 10.3% in men) and a lower incidence of AF (0.16% per year in women compared with men 0.38% per year).^5^ Interestingly, the Huawei heart study showed similar rates in irregular heart rate notifications, but a higher rate of digitally detected AF in women.^9^ However, the participation rate of females in the Huawei heart study was very low (13%).

Compared with previous digital AF screening studies in which the primary endpoint was digitally detected AF, in eBRAVE-AF, the primary endpoint was AF detection with initiation of OAC. There was no difference in the frequency of subsequent prescription of OAC after AF diagnosis between men and women in our study. In eBRAVE-AF, OAC was initiated at the discretion of the treating physicians based on CHA_2_DS_2_-VASc Score, in accordance with the ESC-Guideline recommendations valid at the time of the study.^6^ Recently, a large population-based cohort study in patients with low apparent stroke risk demonstrated a similar impact of AF in women and men for incident stroke,^27^ leading to a change in ESC-Guideline recommendations to use the CHA_2_DS_2_-VA (excluding gender) to assist in decisions on OAC therapy.^28^ We believe that intermittent and continuous screening have different impact on the indication for OAC. Both European and American guidelines emphasize the need to further investigate the optimal AF-burden cut-off value to initiate OAC.^28,29^ Our results from the eBRAVE-AF trial contribute to this goal and will be useful for planning of future trials.

In eBRAVE-AF, we used a cross-over design, which has several advantages and disadvantages. One of the main advantages of the cross-over design is the higher willingness of invited individuals to participate in the study and the lower drop-out rate among participants randomized to the usual care group. In addition, while the primary endpoint was assessed in the first 6-month phase of the trial the cross-over design provides more power for secondary analyses. The main disadvantage of the cross-over design is the high level of awareness among those randomized to the usual care group, which may lead to an overestimation of the primary endpoint rate in this group. Another disadvantage is the creation of a window period between invitation and implementation of the cross-over, which may impede the classification of endpoints between treatment phases.

Τhis eBRAVE-AF substudy can be used as a backbone for planning future AF-related digital trials. Digital trials should take advantage of their main strength, i.e. the use of modern digital tools, such as on-the-fly randomisation tools with stratification by sex, dynamic event-driven sample size tools with automatic event-based shaping of the sex distribution in order to provide conclusive results for both sexes.

Our study has several limitations. First, while our results demonstrated a significant effect of digital AF screening over usual care in detecting treatment-relevant AF in men, there was a pre-selection of male participants and the participation rate was 8%, which is comparable to other direct-to-participant trials. Second, the overall number of events in female participants was low limiting the statistical power for evaluation of sex-specific interactions. Third, in our study, we used an intermittent, active, smartphone-based screening, which differs from continuous, passive, smartwatch-based screening, which is thought to result in better compliance and increased AF detection sensitivity. Fourth, the cross-over design of the study is likely to have led to an overestimation of the primary endpoint in usual care arm due to increased awareness among study participants. Fifth, this was a siteless trial. Therefore, no specific biomarkers, such as NT-proBNP have been available for study participants. Given that NT-proBNP has shown greater prognostic value in women than men,^30^ using this biomarker to identify high-risk females who may particularly benefit from digital screening could be a promising approach. Finally, our study does not allow conclusions about the implications of digital AF screening on ischaemic events and MACCE. Τhe net clinical benefit of OAC in patients with AF detected by intermittent PPG measurements, as well as the role of female sex as a risk factor for stroke in these patients has to be further elucidated.

In conclusion, a large-scale clinical trial, comparing digital screening to usual care for detecting AF requiring OAC showed that men had a slightly but significantly higher willingness of study participation and that digital AF screening was effective in men. While digital screening resulted in numerically higher detection rates of AF treated with OAC in women, this effect was not statistically significant, most likely due to limited statistical power as a result of overall lower event rates and underrepresentation of female study participants in our study. However, since women have a higher risk for AF-associated complications than men, screening for AF in women is particularly relevant.^5^ The differential efficacy of digital screening between sexes is not only dependent on the incidence of AF in the sexes, but also on other parameters, such as compliance and AF-burden. To our best knowledge, this is a novel observation that has not been previously described, and we consider it an important finding. When planning future AF screening trials, an anticipated lower AF rate in women and optimized pre-selection of female participants should be considered to ensure adequate statistical power. Incorporating sex-adapted biomarkers like NT-proBNP, a robust AF predictor with greater prognostic value in women compared with men, could help identify high-risk females who may benefit from digital screening. Additionally, a continuous, passive PPG monitoring through wearables and AI-driven analysis of PPG signals may further enhance screening accuracy.

## Supplementary Material

ztaf071_Supplementary_Data

## Data Availability

The data underlying this article will be shared on reasonable request to the corresponding author.

## References

[1] Roth GA, Mensah GA, Johnson CO, Addolorato G, Ammirati E, Baddour LM, et al Global burden of cardiovascular diseases and risk factors, 1990–2019: update from the GBD 2019 study. J Am Coll Cardiol 2020;76:2982–3021. 33309175 10.1016/j.jacc.2020.11.010 PMC7755038

[2] Dagres N, Nieuwlaat R, Vardas PE, Andresen D, Lévy S, Cobbe S, et al Gender-related differences in presentation, treatment, and outcome of patients with atrial fibrillation in Europe: a report from the euro heart survey on atrial fibrillation. J Am Coll Cardiol 2007;49:572–577. 17276181 10.1016/j.jacc.2006.10.047

[3] Piccini JP, Simon DN, Steinberg BA, Thomas L, Allen LA, Fonarow GC, et al Differences in clinical and functional outcomes of atrial fibrillation in women and men: two-year results from the ORBIT-AF registry. JAMA Cardiol 2016;1:282–291. 27438106 10.1001/jamacardio.2016.0529

[4] Schnabel RB, Pecen L, Ojeda FM, Lucerna M, Rzayeva N, Blankenberg S, et al Gender differences in clinical presentation and 1-year outcomes in atrial fibrillation. Heart 2017;103:1024–1030. 28228467 10.1136/heartjnl-2016-310406 PMC5529986

[5] Tian XT, Xu YJ, Yang YQ. Gender differences in arrhythmias: focused on atrial fibrillation. J Cardiovasc Transl Res 2020;13:85–96. 31637585 10.1007/s12265-019-09918-w

[6] Hindricks G, Potpara T, Dagres N, Arbelo E, Bax JJ, Blomström-Lundqvist C, et al 2020 ESC guidelines for the diagnosis and management of atrial fibrillation developed in collaboration with the European Association for Cardio-Thoracic Surgery (EACTS): the task force for the diagnosis and management of atrial fibrillation of the European Society of Cardiology (ESC) developed with the special contribution of the European Heart Rhythm Association (EHRA) of the ESC. Eur Heart J 2021;42:373–498. 32860505 10.1093/eurheartj/ehaa612

[7] Lubitz SA, Faranesh AZ, Selvaggi C, Atlas SJ, McManus DD, Singer DE, et al Detection of atrial fibrillation in a large population using wearable devices: the fitbit heart study. Circulation 2022;146:1415–1424. 36148649 10.1161/CIRCULATIONAHA.122.060291 PMC9640290

[8] Perez MV, Mahaffey KW, Hedlin H, Rumsfeld JS, Garcia A, Ferris T, et al Large-scale assessment of a smartwatch to identify atrial fibrillation. N Engl J Med 2019;381:1909–1917. 31722151 10.1056/NEJMoa1901183 PMC8112605

[9] Guo Y, Lane DA, Wang L, Zhang H, Wang H, Zhang W, et al Mobile health technology to improve care for patients with atrial fibrillation. J Am Coll Cardiol 2020;75:1523–1534. 32241367 10.1016/j.jacc.2020.01.052

[10] Rizas KD, Freyer L, Sappler N, von Stülpnagel L, Spielbichler P, Krasniqi A, et al Smartphone-based screening for atrial fibrillation: a pragmatic randomized clinical trial. Nat Med 2022;28:1823–1830. 36031651 10.1038/s41591-022-01979-w

[11] Freyer L, von Stülpnagel L, Spielbichler P, Sappler N, Wenner F, Schreinlechner M, et al Rationale and design of a digital trial using smartphones to detect subclinical atrial fibrillation in a population at risk: the eHealth-based bavarian alternative detection of atrial fibrillation (eBRAVE-AF) trial. Am Heart J 2021;241:26–34. 34252387 10.1016/j.ahj.2021.06.008

[12] Soliman EZ, Lopez F, O'Neal WT, Chen LY, Bengtson L, Zhang ZM, et al Atrial fibrillation and risk of ST-segment-elevation versus non-ST-segment-elevation myocardial infarction: the atherosclerosis risk in communities (ARIC) study. Circulation 2015;131:1843–1850. 25918127 10.1161/CIRCULATIONAHA.114.014145 PMC4447576

[13] Emdin CA, Wong CX, Hsiao AJ, Altman DG, Peters SA, Woodward M, et al Atrial fibrillation as risk factor for cardiovascular disease and death in women compared with men: systematic review and meta-analysis of cohort studies. Bmj 2016;532:h7013. 26786546 10.1136/bmj.h7013 PMC5482349

[14] Shandhi MMH, Singh K, Janson N, Ashar P, Singh G, Lu B, et al Assessment of ownership of smart devices and the acceptability of digital health data sharing. NPJ Digit Med 2024;7:44. 38388660 10.1038/s41746-024-01030-x PMC10883993

[15] Statistisches Bundesamt . Health insurance coverage. https://www.destatis.de/EN/Themes/Labour/Labour-Market/Quality-Employment/Dimension2/2_4_HealthInsuranceCoverage.html.

[16] Connelly PJ, Currie G, Delles C. Sex differences in the prevalence, outcomes and management of hypertension. Curr Hypertens Rep 2022;24:185–192. 35254589 10.1007/s11906-022-01183-8 PMC9239955

[17] Kautzky-Willer A, Leutner M, Harreiter J. Sex differences in type 2 diabetes. Diabetologia 2023;66:986–1002. 36897358 10.1007/s00125-023-05891-x PMC10163139

[18] Carandang R, Seshadri S, Beiser A, Kelly-Hayes M, Kase CS, Kannel WB, et al Trends in incidence, lifetime risk, severity, and 30-day mortality of stroke over the past 50 years. JAMA 2006;296:2939–2946. 17190894 10.1001/jama.296.24.2939

[19] Reeves MJ, Bushnell CD, Howard G, Gargano JW, Duncan PW, Lynch G, et al Sex differences in stroke: epidemiology, clinical presentation, medical care, and outcomes. Lancet Neurol 2008;7:915–926. 18722812 10.1016/S1474-4422(08)70193-5 PMC2665267

[20] George J, Rapsomaniki E, Pujades-Rodriguez M, Shah AD, Denaxas S, Herrett E, et al How does cardiovascular disease first present in women and men? Incidence of 12 cardiovascular diseases in a contemporary cohort of 1,937,360 people. Circulation 2015;132:1320–1328. 26330414 10.1161/CIRCULATIONAHA.114.013797 PMC4590518

[21] Steinhubl SR, Waalen J, Edwards AM, Ariniello LM, Mehta RR, Ebner GS, et al Effect of a home-based wearable continuous ECG monitoring patch on detection of undiagnosed atrial fibrillation: the mSToPS randomized clinical trial. JAMA 2018;320:146–155. 29998336 10.1001/jama.2018.8102 PMC6583518

[22] Sandberg EL, Halvorsen S, Berge T, Grimsmo J, Atar D, Fensli R, et al Fully digital self-screening for atrial fibrillation with patch electrocardiogram. Europace 2023;25:euad075. 36945146 10.1093/europace/euad075 PMC10227758

[23] Sandberg EL, Halvorsen S, Berge T, Grimsmo J, Atar D, Leangen Grenne B, et al Digital recruitment and compliance to treatment recommendations in the Norwegian atrial fibrillation self-screening pilot study. Eur Heart J Digit Health 2024;5:371–378. 38774377 10.1093/ehjdh/ztae026 PMC11104466

[24] Svennberg E, Engdahl J, Al-Khalili F, Friberg L, Frykman V, Rosenqvist M. Mass screening for untreated atrial fibrillation: the STROKESTOP study. Circulation 2015;131:2176–2184. 25910800 10.1161/CIRCULATIONAHA.114.014343

[25] Gudmundsdottir KK, Holmen A, Fredriksson T, Svennberg E, Al-Khalili F, Engdahl J, et al Decentralising atrial fibrillation screening to overcome socio-demographic inequalities in uptake in STROKESTOP II. J Med Screen 2021;28:3–9. 32228146 10.1177/0969141320908316 PMC7905746

[26] Paolillo EW, Lee SY, VandeBunte A, Djukic N, Fonseca C, Kramer JH, et al Wearable use in an observational study among older adults: adherence, feasibility, and effects of clinicodemographic factors. Front Digit Health 2022;4:884208. 35754462 10.3389/fdgth.2022.884208 PMC9231611

[27] Mobley AR, Subramanian A, Champsi A, Wang X, Myles P, McGreavy P, et al Thromboembolic events and vascular dementia in patients with atrial fibrillation and low apparent stroke risk. Nat Med 2024;30:2288–2294. 38839900 10.1038/s41591-024-03049-9 PMC11333279

[28] Van Gelder IC, Rienstra M, Bunting KV, Casado-Arroyo R, Caso V, Crijns HJGM, et al 2024 ESC guidelines for the management of atrial fibrillation developed in collaboration with the European Association for Cardio-Thoracic Surgery (EACTS). Eur Heart J 2024;45:3314–3414. 39210723 10.1093/eurheartj/ehae176

[29] Joglar JA, Chung MK, Armbruster AL, Benjamin EJ, Chyou JY, Cronin EM, et al 2023 ACC/AHA/ACCP/HRS guideline for the diagnosis and management of atrial fibrillation: a report of the American College of Cardiology/American Heart Association joint committee on clinical practice guidelines. Circulation 2024;149:e1–e156. 38033089 10.1161/CIR.0000000000001193 PMC11095842

[30] Patton KK, Heckbert SR, Alonso A, Bahrami H, Lima JA, Burke G, et al N-terminal pro-B-type natriuretic peptide as a predictor of incident atrial fibrillation in the multi-ethnic study of atherosclerosis: the effects of age, sex and ethnicity. Heart 2013;99:1832–1836. 24131775 10.1136/heartjnl-2013-304724

