# Supplementary material for: Gender specific aspects of digital screening for atrial fibrillation: insights from the randomized eBRAVE-AF trial
Source: Eur Heart J Digit Health. 2025 Jun 19;6(5):1015–23. doi: 10.1093/ehjdh/ztaf071 (PMC12450515; doi:10.1093/ehjdh/ztaf071)
Supplement: ztaf071_Supplementary_Data [file ztaf071_supplementary_data.docx]

**Supplementary Table 1:** Uni-and multivariate analysis for incident atrial fibrillation with OAC

| **Uni- and multivariate analysis for primary endpoint (AF+OAC)** | | | | | | |
| --- | --- | --- | --- | --- | --- | --- |
|  | **overall** | | **digital screening** | | **usual care** | |
|  | **univariate**  HR (95% CI); p-value | **multivariate**  HR (95% CI); p-value | **univariate**  HR (95% CI); p-value | **multivariate**  HR (95% CI); p-value | **univariate**  HR (95% CI); p-value | **multivariate**  HR (95% CI); p-value |
| **male sex** | 1.74 (1.08-2.82);p=0.023 | 1.58 (0.97-2.56);p=0.067 | 1.92 (1.07-3.42);p=0.029 | 1.71 (0.95-3.06);p=0.07 | 1.41 (0.60-3.30);p=0.428 |  |
| **age** | 1.09 (1.06-1.11);p<0.001 | 1.08 (1.05-1.11);p<0.001 | 1.09 (1.06-1.12);p<0.001 | 1.08 (1.05-1.12);p<0.001 | 1.08 (1.03-1.13); p=0.002 | 1.07 (1.02-1.12);p=0.007 |
| **diabetes** | 1.27 (0.74-2.16);p=0.383 |  | 1.07 (0.55-2.09);p=0.842 |  | 1.80 (0.83-4.42); p=0.200 |  |
| **hypertension** | 1.41 (0.91-2.17);p=0.124 |  | 1.91 (1.10-3.32);p=0.023 | 1.75 (1.02-3.09);p=0.043 | 0.75 (0.36-1.57);p=0.447 |  |
| **stroke** | 1.02 (0.45-2.33);p=0.962 |  | 1.20 (0.48-2.97),p=0.700 |  | 1.70 (0.08-4.32);p=0.601 |  |
| **heart failure** | 1.24 (0.50-3.04);p=0.642 |  | 1.00 (0.31-3.16);p=0.994 |  | 1.84 (0.44-7.74);p=0.406 |  |
| **vascular disease** | 1.58 (1.00-2.50);p=0.049 | 1.12 (0.70-1.80);p=0.632 | 1.21 (0.68-2.17);p=0.517 |  | 2.73 (1.27-5.86);p=0.010 | 2.20 (1.01-4.80);p=0.048 |

HR, hazard ratio

**Supplementary table 2:** Uni-and multivariate analysis for incident atrial fibrillation

| **Uni- and multivariate analysis for atrial fibrillation** | | | | | | |
| --- | --- | --- | --- | --- | --- | --- |
|  | **overall** | | **digital screening** | | **usual care** | |
|  | **univariate**  HR (95% CI); p-value | **multivariate**  HR (95% CI); p-value | **univariate**  HR (95% CI); p-value | **multivariate**  HR (95% CI); p-value | **univariate**  HR (95% CI); p-value | **multivariate**  HR (95% CI); p-value |
| **male sex** | 1.71 (1.13-2.60);p=0.011 | 1.61 (1.06-2.45);p=0.025 | 1.78 (1.07-2.93);p=0.026 | 1.63 (0.98-2.70);p=0.058 | 1.60 (0.76-3.35);p=0.213 |  |
| **age** | 1.06 (1.04-1.09);p<0.001 | 1.06 (1.04-1.08);p<0.001 | 1.06 (1.04-1.09);p<0.001 | 1.06 (1.03-1.09);p<0.001 | 1.05 (1.02-1.10);p=0.005 | 1.06 (1.01-1.10);p=0.010 |
| **diabetes** | 1.34 (0.85-2.12);p=0.210 |  | 1.19 (0.68-2.11);p=0.543 |  | 1.68 (0.78-3.64);p=0.188 |  |
| **hypertension** | 1.13 (0.78-1.62);p=0.525 |  | 1.54 (0.97-2.45);p=0.069 | 1.45 (0.91-2.31);p=0.118 | 1.64 (0.33-1.13);p=0.116 |  |
| **stroke** | 0.90 (0.42-1.92);p=0.782 |  | 1.12 (0.49-2.57);p=0.786 |  | 2.44 (0.56-2.98);p=0.378 |  |
| **heart failure** | 1.13 (0.50-2.55);p=0.776 |  | 1.29 (0.25-2.45);p=0.665 |  | 1.96 (0.61-6.36);p=0.261 |  |
| **vascular disease** | 1.24 (0.81-1.90);p=0.332 |  | 0.98 (0.56-1.71);p=0.946 |  | 1.90 (0.95-3.78);p=0.069 | 1.59 (0.79-3.22);p=0.193 |

HR, hazard ratio

**Supplementary Table 3:**

|  | **Females without AF**  **(N=1.697)** | **Females**  **with AF**  **(N=28)** | ***p*-value** | **Males**  **without AF**  **(N=3.720)** | **Males**  **with AF**  **(N=106)** | ***p*-value** |
| --- | --- | --- | --- | --- | --- | --- |
| **Characteristics** | | | | | | |
| Age (years) | 65 (59-70) | 72 (66-77) | <0.001 | 66 (60-71) | 69 (64-75) | <0.001 |
| BMI (kg/m²) | 25 (23-29) | 24 (23-29) | 0.874 | 27 (25-30) | 27 (25-30) | 0.664 |
| CHA_2_DS_2_-VASc Score | 3 (3-4) | 4 (3-4) | 0.032 | 3 (2-3) | 3 (2-3) | 0.018 |
| Coronary heart disease | 118 (7.0) | 2 (7.1) | 1.000 | 675 (18.1) | 23 (21.7) | 0.420 |
| Previous MI | 32 (1.9) | 2 (7.1) | 0.194 | 317 (8.5) | 11 (10.4) | 0.619 |
| Heart failure | 77 (4.5) | 3 (10.7) | 0.276 | 143 (3.8) | 3 (2.8) | 0.779 |
| Valvular heart disease | 124 (7.3) | 5 (17.9) | 0.081 | 208 (5.6) | 9 (8.5) | 0.289 |
| History of stroke | 62 (3.7) | 3 (10.7) | 0.148 | 251 (6.7) | 4 (3.8) | 0.331 |
| COPD | 5 (0.3) | 0 (0.0) | 1.000 | 9 (0.2) | 0 (0.0) | 1.000 |
| Chronic kidney disease | 6 (0.4) | 0 (0.0) | 1.000 | 9 (0.2) | 1 (0.9) | 0.667 |
| Diabetes | 149 (8.8) | 2 (7.1) | 1.000 | 546 (14.7) | 20 (18.9) | 0.290 |
| Hypertension | 1043 (61.5) | 18 (64.3) | 0.913 | 2495 (67.1) | 73 (68.9) | 0.780 |
| History of loss of consciousness | 101 (6.0) | 1 (3.6) | 0.900 | 173 (4.7) | 4 (3.8) | 0.849 |
| History of bleeding | 55 (3.2) | 1 (3.6) | 1.000 | 155 (4.2) | 6 (5.7) | 0.661 |
| History of thromboembolism | 89 (5.2) | 3 (10.7) | 0.393 | 331 (8.9) | 11 (10.4) | 0.724 |
| **Symptoms** | | | | | | |
| Dyspnea or angina | 477 (28.1) | 12 (42.9) | 0.132 | 617 (16.6) | 26 (24.5) | 0.043 |
| Palpitations | 491 (28.9) | 13 (46.4) | 0.070 | 586 (15.8) | 44 (41.5) | <0.001 |
| **Medication** | | | | | | |
| ACE inhibitor or ARB | 227 (13.4) | 1 (3.6) | 0.215 | 493 (13.3) | 9 (8.5) | 0.198 |
| Beta-blocker | 104 (6.1) | 1 (3.6) | 0.869 | 55 (1.5) | 4 (3.8) | 0.136 |
| Aspirin | 12 (0.7) | 0 (0.0) | 1.000 | 25 (0.7) | 0 (0.0) | 0.814 |
| Other antiplatelet agents | 4 (0.2) | 0 (0.0) | 1.000 | 3 (0.1) | 0 (0.0) | 1.000 |

**Supplementary Table 3:** Baseline characteristics stratified by sex and detection of AF during the course of the trial.

**Supplementary Table 4:**

|  | **Women** | **Men** | ***p*-value** |
| --- | --- | --- | --- |
| **AF leading to OAC** | 21 (100%) | 81 (100%) |  |
| Persistent | 3 (14%) | 22 (27%) | *0.176 |
| Paroxysmal | 17 (81%) | 51 (63%) |  |
| Max duration (h) | 5 (1-21) | 12 (5-52) | 0.243 |
| AF burden (%) | 3.9 (1.3-13.7) | 6.9 (2.3-54.7) | 0.388 |
| Not classified | 1 (5%) | 8 (10%) |  |

**Supplementary Table 4:** AF-characteristics stratified by sex; *The chi-square test was used for comparison of types of AF between groups.
